# CXCL-10 in Cerebrospinal Fluid Detects Neuroinflammation in HTLV-1-Associated Myelopathy with High Accuracy

**DOI:** 10.3390/v17010089

**Published:** 2025-01-12

**Authors:** Samya Jezine Da Silva, Mauro Jorge Cabral-Castro, Luiz Claudio Faria, Carolina Rosadas, Maria Fernanda Lopes de Araújo, Ana Caroline Soares Dutra, Yoshihisa Yamano, Graham Taylor, Marzia Puccioni-Sohler

**Affiliations:** 1 Programa de Pós-Graduação em Doenças Infecciosas e Parasitárias, Faculdade de Medicina, Universidade Federal do Rio de Janeiro, Rio de Janeiro 21941-913, Brazil; 2 Departamento de Patologia—Programa de Pós-Graduação em Patologia, Faculdade de Medicina, Universidade Federal Fluminense, Niterói 24070-090, Brazil; maurojorgerj@gmail.com; 3 Laboratório de Líquido Cefalorraquidiano, Hospital Universitário Clementino Fraga Filho, Universidade Federal do Rio de Janeiro, Rio de Janeiro 21941-913, Brazil; luizclaudio@hucff.ufrj.br; 4 Section of Virology, Department of Infectious Disease, Imperial College, London W2 1PG, UK; c.rosadas-de-oliveira@imperial.ac.uk (C.R.); g.p.taylor@imperial.ac.uk (G.T.); 5 Escola de Medicina e Cirurgia, Universidade Federal do Estado do Rio de Janeiro, Rio de Janeiro 20270-004, Brazil; fernandaraujo@edu.unirio.br (M.F.L.d.A.); anadutra@edu.unirio.br (A.C.S.D.); 6 Department of Neurology, St. Marianna University School of Medicine, Kawasaki 216-8512, Japan; yyamano@marianna-u.ac.jp

**Keywords:** accuracy, diagnosis, HTLV-1-associated myelopathy, cerebrospinal fluid, neopterin, CXCL-10, biomarker

## Abstract

Background and Objectives: HTLV-1-associated myelopathy (HAM) is a chronic progressive inflammatory disease of the spinal cord. This study assesses the diagnostic accuracy of the neuroinflammatory biomarkers neopterin and cysteine-X-cysteine motif chemokine ligand 10 (CXCL-10) in cerebrospinal fluid (CSF) for HAM. Methods: CSF samples from 75 patients with neurological disorders—33 with HAM (Group A), 19 HTLV-1-seronegative with other neuroinflammatory diseases (Group B), and 23 HTLV-1-seronegative with non-neuroinflammatory diseases (Group C)—were retrospectively evaluated. CSF examination included routine analysis, neopterin, and CXCL-10. The diagnostic potential of the biomarkers was evaluated using receiver operating characteristic curves. Results: Higher white cell counts and concentrations of protein, neopterin, and CXCL-10 in CSF were detected in group A (patients with HAM) and group B (*p* < 0.05). Neopterin showed good accuracy for HAM (A) (cut-off 15 nmol/L, 80% sensitivity, 74% specificity) and other neuroinflammation (group B) (cut-off 20 nmol/L, 79% sensitivity, 83% specificity). CXCL-10 demonstrated the highest accuracy in both groups, with Group A (cut-off 110 pg/mL, 97% sensitivity, 96% specificity) and Group B (cut-off 220 pg/mL, 100% sensitivity, 100% specificity). Conclusions: Neopterin and CXCL-10 in CSF are accurate biomarkers for detecting neuroinflammation, including HAM. CXCL-10, in particular, is the superior biomarker for both chronic and acute neuroinflammatory diseases.

## 1. Introduction

Human T-cell leukemia virus type 1 (HTLV-1) was the first human retrovirus isolated in humans [1]. There is a previous estimation of 5–10 million HTLV-1-infected individuals worldwide [2]. In fact, the number of people who have been infected since 1980 has not been estimated but must be significantly higher. HTLV-1 is transmitted through mother-to-child contact, sexual intercourse, contaminated blood products, and organ transplantation [3]. HTLV-1 is endemic in southwestern Japan, sub-Saharan Africa, South America, the Caribbean, the Middle East, and Australo-Melanesia [2].

Pro hypothesis, the minimum of 2.25%, but with an upper bound of no less than 10%, of the infected people are predisposed to developing diseases: cutaneous T-cell lymphoma (ATL) alone represents 2–6%, and HTLV-1-associated myelopathy (HAM) represents 0.25% (Japan) to 3.8%, plus other inflammatory conditions [4,5]. While ATL is a carcinogenic process, HTLV-1-associated myelopathy (HAM) is an inflammatory demyelination disease of the spinal cord caused by the virus [3,6].

HAM is characterized by progressive weakness of the lower extremities, and urinary and sensory disturbances [7]. The disease progresses with significant disability. Patients become dependent on wheelchairs or bedridden [8]. The risk of developing HAM is associated with the duration of infection, high proviral load, and host immunogenetic responses [9].

HTLV-1 mainly targets CD4+ cells, but it also infects other cell types, including CD8+ T cells, dendritic cells, monocytes, and endothelial cells [8,10]. The progression of HAM may be influenced by the complex immune responses triggered by the interaction between HTLV-1-infected cells and the host immune system. Activated HTLV-1-infected CD4+ T cells cross the blood–brain and blood–cerebrospinal fluid barriers into the central nervous system (CNS), where they express viral antigens and produce proinflammatory cytokines, chemokines, and other neuroinflammatory mediators [10]. These include interferon-γ (IFN-γ), tumor necrosis factor-α (TNF-α), and interleukin-1β (IL-1β) [6,8].

In HAM, the cytokine IFN-γ stimulates astrocytes to produce high levels of the cysteine-X-cysteine motif chemokine ligand 10 (CXCL-10), a chemokine that recruits more infected T cells, promoting a continuous loop of infiltration of inflammatory cells into the CNS, causing its damage [6,8]. Neopterin is another biomarker of cellular immune activation synthesized by macrophages/monocytes and astrocytes, under stimulation with IFN- γ, and is strongly correlated with the HAM progression [8,11,12].

CXCL-10 and neopterin have been extensively studied in various neuroinflammatory diseases [13,14,15]. Their potential use as biomarkers for monitoring disease progression and treatment response in HAM, especially regarding corticosteroid therapy, has gained attention [16,17]. Nonetheless, further exploration is warranted to establish their diagnostic accuracy [8].

Therefore, understanding the role of the cellular immune response to HTLV-1 in HAM underscores the importance of identifying sensitive neuroinflammatory biomarkers for early disease diagnosis. This study aims to evaluate the diagnostic accuracy of neopterin and CXCL-10 in cerebrospinal fluid (CSF) for detecting neuroinflammation in HAM.

## 2. Subjects and Methods

### 2.1. Specimens Studied

This retrospective cross-sectional study analyzed stored leftover cerebrospinal fluid (CSF) samples obtained from routine diagnostic testing of 33 patients with HAM [7] and, as control, 42 CSF specimens of HTLV-1-uninfected individuals were categorized into two groups. Group B consisted of 19 inflammatory CSF samples (white cell count > 4 cells/mm^3^, protein > 45 mg/dL) from 19 patients with acute neuroinflammatory diseases (Guillain–Barré syndrome, neuromyelitis optica, viral encephalitis, bacterial and fungal meningitis, herpetic myeloradiculitis), and group C included 23 non-inflammatory CSF samples (white cell count ≤ 4 cells/mm^3^, protein ≤ 45 mg/dL) from patients with non-neuroinflammatory conditions (dementia, neoplasia, idiopathic intracranial hypertension, cerebrovascular disease). The progression of HAM was classified as rapid, slow, or very slow based on the disease activity classification according to Yamano and Sato (2012) [10]. In group A, all patients tested negative for other infectious agents, such as human immunodeficiency virus-1 (HIV-1) and hepatitis C virus (HCV) and syphilis.

### 2.2. HTLV-1/2 Antibody Tests

The serum and CSF samples were screened for anti-HTLV-1/2 antibodies using the enzyme-linked immunosorbent assay (ELISA) (Murex HTLV-I+II; DiaSorin, Dartford, UK). Serological confirmation was performed by Western blot (HTLV BLOT 2.4-Genelabs Diagnostics, Science Park, Singapore).

### 2.3. CSF Routine Analysis

White cell count (by Fuchs–Rosenthal chamber), protein concentration (pyrogallol red method), glucose/lactate (enzymatic method), and VDRL were routinely performed by the pathology services, along with microscopy and culture for bacteria, fungi, and mycobacteria.

### 2.4. Neopterin and CXCL-10 Concentrations

Neopterin and CXCL-10 concentrations in CSF were determined by commercial ELISA tests, following the manufacturers’ protocols (IBL International, Hamburg, Germany for neopterin, and Human IP-10 CXCL-10 ELISA, Invitrogen, Carlsbad, CA, USA for CXCL-10).

### 2.5. HTLV-1 Proviral Load

Viral DNA was extracted from peripheral blood mononuclear cells (PBMC) using the commercial PurelLink Viral RNA/DNA Mini Kit (Invitrogen, CA, USA). HTLV-1 proviral load (PVL) was assessed using real-time PCR [18].

### 2.6. Statistical Analysis

Age was expressed as the mean ± standard deviation (SD) and compared using one-way ANOVA. Categorical variables, such as gender, were presented as frequency (n) and percentage (%) and compared using the chi-square or Fisher’s exact test (*p* ≤ 0.05 for statistical significance). Numerical variables, including time of symptoms, white cell count, protein, neopterin, and CXCL-10 concentrations, were expressed as the median and interquartile range (IQR, Q1-Q3) and compared using the Kruskal–Wallis ANOVA test for three-group comparisons. When significant differences were found (*p* ≤ 0.05), Dunn’s test was employed to distinguish between the groups.

Receiver operating characteristic (ROC) curve analysis was used to calculate the AUC (area under the curve) and the best cut-off values for neopterin and CXCL-10 for determining the points of optimal sensitivity and specificity among the three groups (HAM, and inflammatory compared to non-inflammatory). Statistical analyses were performed using GraphPad Prism version 8 (GraphPad Software, La Jolla, CA, USA). Statistical significance was set at *p* ≤ 0.05.

The Spearman test was used to calculate the correlation coefficient (r) among the numerical variables. The following reference values for the Spearman test were adopted: r ≥ 0.70 for very strong correlation, r = 0.40 to 0.69 for strong correlation, r = 0.30 to 0.39 for moderate correlation, r = 0.20 to 0.29 for weak correlation, and r = 0.01 to 0.19 for negligible correlation or non-correlation. Statistical significance was set at *p* ≤ 0.05.

## 3. Results

### 3.1. Characteristics of HAM Patients

Among the 33 patients with HAM (Group A), the mean ± SD age was 52 ± 11.3 years, with a female predominance (60.6%) (Table 1). The progression of the disease was classified as rapid progression in 51.5% (17/33) of the cases, slow progression in 39.4% (13/33), and very slow progression in 9.1% (3/33). Clinical outcomes indicated that 36.4% (12/33) required unilateral support, 15.2% (5/33) required bilateral support, 15.2% (5/33) exhibited abnormal gait without support, 3.0% (1/33) showed abnormal gait with support, and 30.3% (10/33) were restricted to wheelchair. The median (IQR) time of symptoms before the lumbar puncture was 5 (1.8–12) years.

### 3.2. Group Characteristics

No significant differences were observed in age (*p* = 0.2), with the trend in gender (*p* = 0.0544) among the three groups (A, B, and C) as determined by the one-way ANOVA and Kruskal–Wallis ANOVA tests. There were no differences in neuroinflammatory biomarker (neopterin and CXCL-10) levels based on gender distribution. The concentrations of CSF inflammatory biomarkers were higher in Group B in comparison to the other groups (*p* < 0.05). The median (IQR)s of white cell count, protein, neopterin, and CXCL-10 in HAM (Group A) were still higher than those in the non-neuroinflammatory (Group C) (*p* < 0.05), and the median (IQR) of HTLV-1 proviral load in CSF was 7.6 (1.57–12.7) copies/mL. Analyses of CSF samples of the three groups are shown in Table 1.

**Figure 1:**
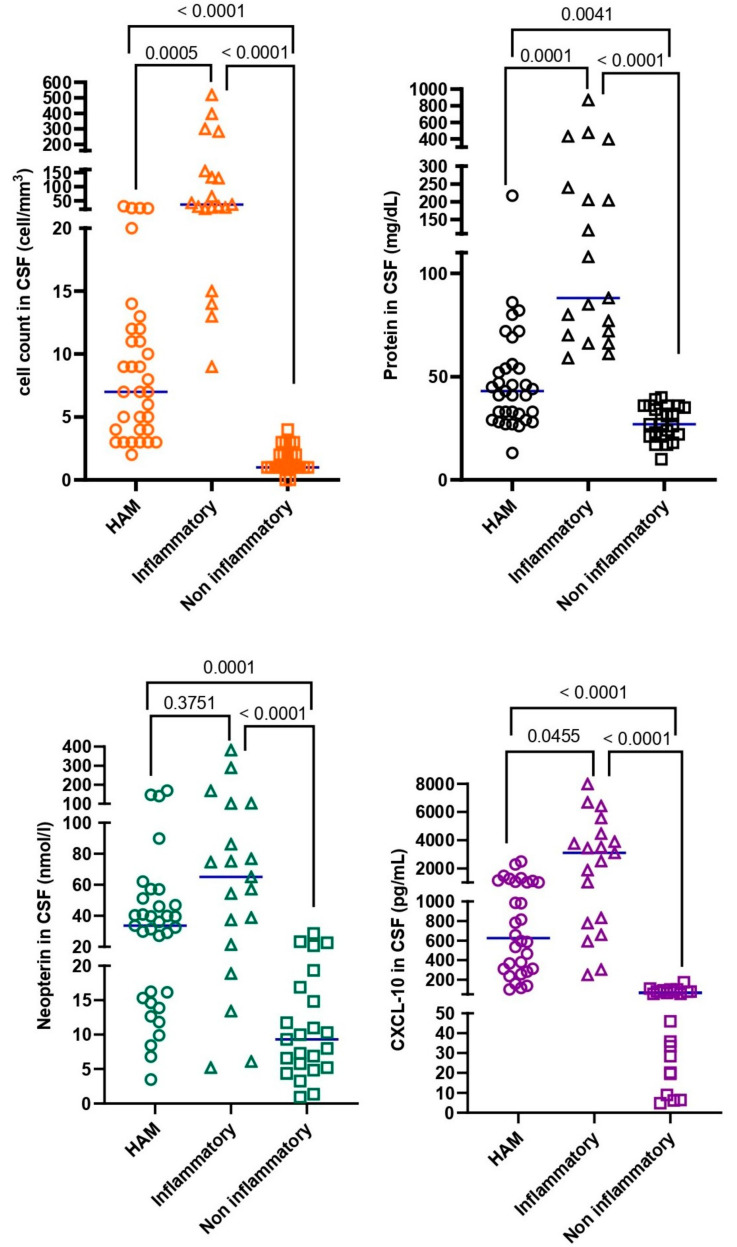
CSF characteristic differences among the three groups (HAM, Inflammatory, and Non-Inflammatory groups). CSF characteristics were compared using Kruskal–Wallis ANOVA, followed by Dunn’s multiple comparison test.

Compared with the non-neuroinflammatory (C) group, Group A had significantly higher CSF protein (*p* = 0.004), neopterin (*p* = 0.0001), white cell count, and CXCL-10 (*p* < 0.0001). Although Group A had a significantly lower white cell count (*p* = 0.0005) and protein concentration (*p* = 0.0001) than Group B, neopterin (*p* > 0.3751) concentrations were similar. CXCL-10 (*p* = 0.0455) concentrations were higher in Group B than in Group A. The differences in CSF biomarkers among the three groups are summarized in Figure 1.

### 3.3. Cut-Off Value, Sensitivity, and Specificity of Neopterin and CXCL-10 in CSF

**Figure 2:**
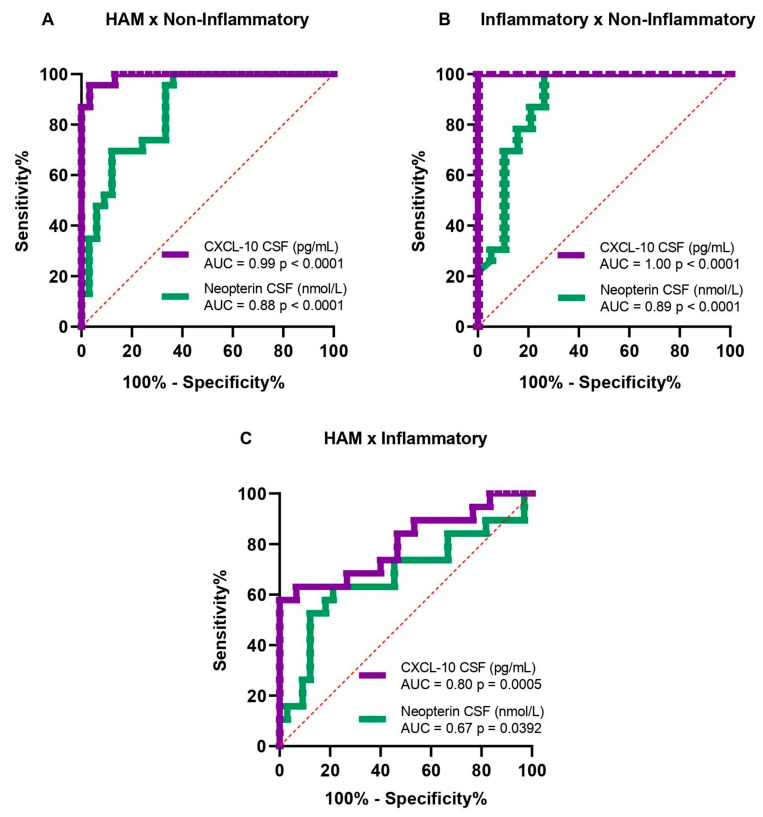
Receiver Operator Characteristic (ROC) curve for neopterin and CXCL-10 in CSF. Differentiating between HAM neuroinflammatory groups relative to the non-neuroinflammatory control group in (**A**,**B**), and between HAM and inflammatory groups in (**C**).

The diagnostic accuracies of neopterin and CXCL-10 among the HAM (group A), neuroinflammatory (group B), and non-neuroinflammatory control (group C) groups are shown in Figure 2. Neopterin demonstrated a good performance, with an area under the curve (AUC) of 0.88 [95% confidence interval (CI): 0.79–0.97; *p* < 0.0001] and a cutoff value of 15 nmol/L, yielding 80% sensitivity and 73.9% specificity to differentiate HAM (group A) from non-neuroinflammatory (group C). Additionally, CXCL-10 exhibited excellent performance, with an AUC of 0.99 [95% CI: 0.97–1.0; *p* < 0.0001] and a cutoff value of 110 pg/mL, resulting in 96.7% sensitivity and 95.7% specificity (Figure 2A).

Comparing the inflammatory (group B) and non-neuroinflammatory (group C) groups, neopterin had very good performance, with an AUC of 0.89 [95% CI: 0.78–1.0; *p* < 0.0001] and a cutoff value of 20 nmol/L, yielding 78.9% sensitivity and 82.6% specificity. Additionally, CXCL-10 showed excellent performance, with an AUC of 1.000 [95% CI: 1.0–1.0; *p* < 0.0001] and a cutoff value of 220 pg/mL, achieving 100% sensitivity and 100% specificity (Figure 2B).

Comparing the HAM (group A) and neuroinflammatory control (group B), the performance was poor for neopterin, with an AUC of 0.67 [95% CI: 0.51–0.84; *p* = 0.0392] and cutoff value of 52.8 nmol/L, yielding 63% sensitivity and 79% specificity. The CXCL-10 demonstrated good performance, with an AUC of 0.8 [95% CI: 0.88–0.94; *p* = 0.0005] and cut-off value of 1015 pg/mL, yielding 68% sensitivity and 73% specificity (Figure 2C).

Table 2 presents the accuracy metrics (sensitivity, specificity, positive predictive value (PPV), negative predictive value (NPV), and percentage of concordance) for the optimal cut-off points of the neopterin and CXCL-10 biomarkers in CSF samples to detect neuroinflammation. These metrics, based on the ROC curve, differentiate the HAM and inflammatory groups from the non-neuroinflammatory (control) group.

The majority of HAM patients (Group A) exhibited pleocytosis (>4 cells/mm^3^), hyperproteinorrachia (>45 mg/dL), elevated concentrations for neopterin (>15 nmol/L), and CXCL-10 (>110 pg/mL) in CSF samples. In Group A, CXCL-10 was the most sensitive biomarker for HAM neuroinflammation diagnosis, indicating an inflammatory profile similar to that of Group B CSF samples (Table 3).

### 3.4. Correlation Between Neopterin and CXCL-10 CSF and Other Neuroinflammatory Biomarkers

We did not find any significant correlation between the proviral load in PBMC and white cell count, protein, neopterin, and CXCL-10 in CSF in the three groups, based on the Spearman test. However, CXCL-10 showed a moderate, though not statistically significant, correlation with protein concentration in CSF samples (r = 0.350; *p* = 0.533). Detailed descriptions of these correlations are provided in Table 4.

## 4. Discussion

HAM is a chronic neuroinflammatory disease of the CNS. Although the pathogenesis remains unclear, it has been shown that HTLV-1-infected cells stimulate the production of proinflammatory cytokines, chemokines, and other neuroinflammatory biomarkers in cells such as macrophages/monocytes and astrocytes, promoting neuroinflammation and neurodegeneration in the CNS [19,20]. This study aimed to evaluate the diagnostic accuracy of the neuroinflammatory biomarkers neopterin and CXCL-10 and determine the optimal cut-off values for HAM diagnosis. We compared CSF samples from HAM patients with samples from patients with acute neuroinflammatory diseases and non-neuroinflammatory diseases. The HAM group showed elevated levels of white cell count, protein, neopterin, and CXCL-10, indicating an inflammatory profile when compared to the non-neuroinflammatory group. The acute neuroinflammatory diseases group showed higher white cell count, protein, neopterin, and CXCL-10 concentrations in CSF than in HAM. Neopterin demonstrated good performance in distinguishing HAM and neuroinflammatory diseases, while CXCL-10 emerged as the most effective biomarker for distinguishing both HAM and acute neuroinflammatory diseases from non-neuroinflammatory conditions. In HAM, there was no significant correlation between the inflammatory biomarkers (white cell count, protein, neopterin, and CXCL-10) in CSF.

Neopterin and CXCL-10 in CSF are locally produced in the CNS and are elevated in cases of brain trauma or CNS infections, such as neuroarboviroses, neuroCOVID-19, and HAM [17,21,22]. These biomarkers have been used for assessing prognosis and evaluating treatment response in HAM patients [8,17,19,23]. In patients with HAM, CXCL-10 and neopterin levels in CSF correlates with Neurofilament Light (NfL), a marker of neuronal damage [24].

Predictive cut-off values for neuroinflammatory biomarkers were defined by Sato et al. (2018) [11] according to the progression of HAM. For neopterin by chromatography test, the cut-off values were ≥44 pmol/L for rapid progression, 6–43 pmol/L for slow progression, and ≤5 pmol/L for very slow progression. For CXCL-10 by cytometric bead array, the cut-off values were ≥4400 pg/mL for rapid progression, 320–4299 pg/mL for slow progression, and IL-6 ≤ 320 pg/mL for very slow progression. The authors used ROC curve analysis to compare groups based on HAM progression (slow vs. very slow and slow vs. very slow vs. control groups, which included asymptomatic carriers and uninfected HTLV-1 patients with non-inflammatory neurological diseases) to determine the cut-off values. We compared CSF samples from HAM and HTLV-1-uninfected non-neuroinflammatory neurological disease patients (control group) to establish cut-off values for neopterin and CXCL-10 based on ELISA tests. In contrast to our study, which focused on diagnostic purposes, Sato et al. (2018) [11] aimed to establish cut-off values for prognostic purposes. The studies are not comparable, considering that we used different methods of analysis.

We observed a higher proportion of HAM patients with rapid progression (51.5%), compared to 13.5% reported by Sato et al. (2018) [11]. Additionally, 39.4% of our patients showed slow progression and 9.1% had very slow progression, whereas Sato et al. (2018) [11] reported 79.8% with slow progression and 6.7% with very slow progression. This indicates a higher proportion of our HAM patients evolved with severe disease progression in a median time of symptoms of 5 (1.8–12) years.

In previous studies using the same method of testing, our group demonstrated that CXCL-10 had also excellent performance in differentiating other acute neuroinflammatory disorders (neuroCOVID-19 and neuroarboviroses) from non-neuroinflammatory conditions [21,22]. Neopterin in CSF had a cut-off value of 11.2 nmol/L, with 67% sensitivity and 63% specificity, while CXCL-10 in CSF showed a cut-off value of 156.5 pg/mL, with 91.7% sensitivity and specificity for neuroarbovirosis (dengue and neurochikungunya) diagnosis [21]. Similarly, for neuroCOVID-19 diagnosis, neopterin demonstrated a cut-off value of 11.9 nmol/L, with 66.7% sensitivity and 42.9% specificity, whereas CXCL-10 had a cut-off value of 174.7 pg/mL, with 95.8% sensitivity and 92.9% specificity. In the same study, serial CSF analysis revealed that one meningoencephalitis case exhibited progressive increases in neopterin and CXCL-10 levels until the patient’s death, while a rhombencephalitis case showed a decrease in biomarker levels until the patient was discharged from the hospital [22]. Here, we identified HAM diagnosis cut-off values for neopterin >15 nmol/L, and the cut-off for CXCL-10 >110 pg/mL. We demonstrated that neopterin had good performance (AUC = 0.88; 80% sensitivity and 74% specificity). CXCL-10 was the most effective biomarker in distinguishing HAM and acute neuroinflammatory diseases (groups A and B) from the non-neuroinflammatory group, showing excellent performance (AUC = 0.99; 97% sensitivity and 96% specificity). In comparison between other acute neuroinflammatory and non-neuroinflammatory diseases, neopterin exhibited good performance as a biomarker, with a cut-off value of 20 nmol/L (79% sensitivity and 83% specificity), and CXCL-10 demonstrated perfect performance, with a cut-off value of 220 pg/mL (100% sensitivity and 100% specificity). These findings confirm the efficacy of neopterin and CXCL-10 as reliable indicators of neuroinflammation.

Our findings indicate that neopterin had better accuracy for HAM disease, with 80% sensitivity and 73.9% specificity, compared to the accuracy reported for acute neuroarboviroses and neuroCOVID-19, based on the same method. Additionally, CXCL-10 exhibited high accuracy for both acute (neuroarboviroses and neuroCOVID-19) and chronic diseases (HAM).

Souza et al. (2021) [17] measured neuroinflammatory and neurodegenerative biomarker (neurofilament light (Nfl) and phosphorylated heavy (pHfH) chains, total tau protein, cellular prion protein (PrPc), inflammatory chemokines, and neopterin) for HAM prognosis, including neopterin and CXCL-10. We found a similar mean age (55.4 ± 13.4 years vs. 51 ± 11.3 years) and a predominance of female patients (61.9% vs. 60.6%). Regarding CSF characteristics, Souza et al. reported a lower proportion of CSF samples with elevated white cell count compared to our findings: 38% vs. 68%, respectively; lower median white cell count and neopterin concentrations, with white cell count at 4 cells/mm^3^ (IQR 1.5–7.5) vs. 7 cells/mm^3^ (IQR 0.4–12) and neopterin at 14.1 nmol/L (IQR 10.48–18.67) vs. 33.7 nmol/L (IQR 15–49). Protein concentrations were similar (43.99 mg/dL ± 12.46 vs. 43 mg/dL, IQR 30.5–55), while CXCL-10 concentrations were higher in their study (1067.5 pg/mL, IQR 830.5–1876.0) vs. 626 pg/mL (IQR 303.2–1069).

The higher proportion of elevated white cell count and neopterin concentrations in our study suggests a more pronounced acute neuroinflammatory response in our HAM patient cohort. This indicates potential differences in disease severity, phases of disease progression, or other underlying conditions that could influence the neuroinflammatory response. Significantly, our cohort is associated with more severe disease.

Tamaki et al. (2019) [16] found that most inflammatory biomarkers, including protein, neopterin, and CXCL-10, decreased after steroid treatment, except for white cell count. Patients who continued therapy showed sustained improvement in both clinical status and levels of CXCL-10 and neopterin over two years. However, only CXCL-10 levels, not neopterin, differed significantly between responders and non-responders in the initial treatment, making CXCL-10 a reliable biomarker for therapy response and prediction in HAM [16].

Some studies have reported gender-related differences in the concentrations of CXCL-12 and CXCL-10 in serum and plasma samples from patients with inflammatory responses due to spinal cord injury and HIV-associated neurocognitive disorder (HAND) [25,26]. In our study, gender difference did not influence the immune response and the biomarker (neopterin and CXCL-10) levels in CSF from patients with HAM and neuroinflammatory and non-neuroinflammatory disorders.

Previous research hypothesized that the pathophysiology and complications of HTLV-1 infection stimulate the synthesis of pro-inflammatory cytokines through the production of CXCL-10 via NF-kB activation, particularly in HTLV-1-infected CNS cells. This mechanism has been demonstrated in other viral infections, such as HCV [27,28]. Studies evaluating the role of NF-κB activation in CXCL-10 production in HTLV-1-infected CNS cells could clarify inflammatory processes associated with HAM and identify new therapeutic targets.

The limitations of the study included the sample size and its retrospective design, which could induce information bias and the lack of control over external variables. However, this last situation was reduced, considering that the HAM cases included in the study are part of a cohort of patients treated for a long time at Hospital Universitário Gaffrée e Guinle (UNIRIO), Rio de Janeiro, Brazil. On the other hand, CSF samples are difficult to obtain since they involve an invasive diagnosis procedure, lumbar puncture.

## 5. Conclusions

In conclusion, CXCL-10 and neopterin in CSF proved to be the most effective biomarker for differentiating HAM from non-neuroinflammatory conditions, with an almost perfect performance and the best accuracy of CXCL-10. The consistent elevation of neopterin and CXCL-10 in HAM patients reinforces their potential as reliable biomarkers for prognosis and treatment evaluation.

## Figures and Tables

**Table 1:** Demographic data and CSF findings in HAM in comparison to the control groups.

	Group A: HAM (n = 33)	Group B: Inflammatory (n = 19)	Group C: Non-Inflammatory (n = 23)	*p* Values
Age (years), mean (±SD)	51.1 (±11.3)	43 (±17.3)	51.8 (±22.2)	0.1759
Female, n (%)	20 (60.6%)	9 (47.4%)	19 (82.6%)	0.0544
**CSF**				
White cell count, median (IQR) (cells/mm^3^)	7 (4–12)	37 (22–155)	1 (1–2)	<0.0001
Protein, median (IQR) (mg/dL)	43 (30.5–55)	88 (70–240)	27 (21–36)	<0.0001
Neopterin, median (IQR) (nmol/L)	33.7 (15–49)	65.1 (21.5–101.4)	9.3 (5.2–16.9)	<0.0001
CXCL-10, median (IQR) (pg/mL)	626 (303.2–1069)	3098 (778.7–4469)	55.2 (20.1–81.4)	<0.0001
**Blood**				
Proviral load, median (IQR) (copies/mL)	7.6 (1.57–12.7)	NA	NA	NA

Age data were expressed as mean and SD and compared using one-way ANOVA. Categorical data (sex) were presented as frequency (n) and percentage (%) and analyzed with the Kruskal–Wallis ANOVA test. Numerical variables were expressed as median and IQR (Q1–Q3). Differences between groups were evaluated using the Kruskal–Wallis ANOVA for four groups (white cell count, protein, neopterin, and CXCL-10). Reference values: white cell count ≤ 4 cells/mm^3^, protein 15–45 mg/dL. Significant differences were reported for *p* ≤ 0.05. CSF: cerebrospinal fluid; CXCL-10: C-X-C motif chemokine ligand 10; HAM: HTLV-1 associated to myelopathy/tropical spastic paraparesis; IQR: Interquartile Range (Q1–Q3); NA: Not Applicable; SD: Standard Deviation.

**Table 2:** Accuracy analysis of the inflammatory biomarkers in different groups.

Accuracy Analysis	Biomarkers	Cut Off	Sensitivity (%)	Specificity (%)	PPV (%)	NPV (%)
**HAM ×** **Non-Inflammatory**	**Neopterin** **(nmol/L)**	15	80.0	73.9	80.0	73.9
	**CXCL-10** **(pg/mL)**	110	96.7	95.7	96.7	95.7
**Inflammatory ×** **Non-Inflammatory**	**Neopterin** **(nmol/L)**	20	78.9	82.6	78.9	82.6
	**CXCL-10** **(pg/mL)**	220	100	100	100	100

This table presents the accuracy analysis of neopterin and CXCL-10 in CSF samples, distinguishing between the different groups (HAM and inflammatory) compared to the non-neuroinflammatory group. The cut-off values are expressed as nmol/L for neopterin and pg/mL for CXCL-10. Sensitivity, specificity, PPV, NPV, and concordance are expressed as percentages. Abbreviations: PPV—Positive Predictive Value; NPV—Negative Predictive Value.

**Table 3:** Proportion of elevated CSF inflammatory biomarkers in the HAM group in comparison to the control groups.

Groups	White Cell Count (>4 Cells/mm^3^)	Protein (>45 mg/dL)	Neopterin (>15 nmol/L)	CXCL-10 (>110 pg/mL)
HAM (%)	69.7% (23/33)	42.4% (14/33)	75.8% (25/33)	97% (32/33)
Inflammatory (%)	100% (19/19)	100% (19/19)	84.2% (16/19)	100% (19/19)
Non-Inflammatory (%)	0% (0/23)	0% (0/23)	26.1% (6/23)	4.3% (1/23)

**Table 4:** Correlation between different inflammatory biomarkers in CSF from the HAM group.

Analysis in CSF	Neopterin (nmol/L)			CXCL-10 (pg/mL)		
	N	R	*p* Value	N	R	*p* Value
White cell count (cells/mm^3^)	33	0.146	0.419	30	-0.067	0.419
Protein (mg/dL)	33	0.113	0.533	**30**	**0.350**	**0.533**
CXCL-10 (pg/mL)	30	0.005	0.979	-	-	-

The correlation coefficient (r) was calculated using the Spearman test. Reference values of Spearman test: r ≥ 0.70 is very strong correlation, r = 0.40 to 0.69 is a strong correlation, r = 0.30 to 0.39 is a moderate correlation, r = 0.20 to 0.29 is a weak correlation, and r = 0.01 to 0.19 is a negligible correlation or non-correlation. *p* < 0.05 values are significant and these are in bold. n is the number of samples analyzed.

## Data Availability

The data collected and analyzed were obtained in accordance with the study methodology and were used for scientific research only after receiving approval from the HUGG/UNIRIO and HUCFF/UFRJ ethics and research committee. Patient confidentiality was strictly maintained. For additional information, please contact the authors.
